# Cricotracheal Adenoid Cystic Carcinoma: Insights Into the Diagnosis and Management of an Uncommon Anatomic Variant

**DOI:** 10.7759/cureus.30686

**Published:** 2022-10-25

**Authors:** Luis Pacheco-Ojeda, Carlos Ríos-Deidán, Stalin Cañizares, Patricia Pontón-Villalba, Edison Moya-Paredes

**Affiliations:** 1 General Surgery, Hospital Metropolitano, Quito, ECU; 2 Otolaryngology, Universidad Central del Ecuador, Quito, ECU; 3 College of Medicine, Universidad San Francisco de Quito, Quito, ECU; 4 General Surgery, Hospital Carlos Andrade Marin, Quito, ECU

**Keywords:** surgical case reports, trachea, larynx, adenoid cystic carcinoma. cancer in the cervix, functional anatomy of the larynx

## Abstract

Adenoid cystic carcinoma (ACC) is the second most common malignant salivary gland tumor and accounts for 30% of minor salivary gland tumors. Its location in the larynx and trachea are rare. We present the case of a 45-year-old healthy male whose MRI revealed a posterior endoluminal tumor that invaded the posteroinferior perichondrium of the cricoid lamina and displaced the hypopharynx and esophagus. A left-limited cervical surgical exploration and an intraluminal incisional biopsy through the tracheostomy space were performed by another surgical team. The pathological study reported an ACC, T4aN0M0, stage IVA tumor. Then, a circular tracheal resection and an excision of the inferior part of the posterior cricoid lamina were carried out. The macroscopic study showed a lesion, 3cm long, 2.2cm wide, and 1cm thick, located at the posterior wall of the cricoid cartilage and proximal trachea. Only the upper margin was compromised. Microscopically, the tumor showed tubular, solid, cribriform, and trabecular patterns. One and a half years after surgery, the patient still has bilateral vocal cord mobility and normal speech. It is clear that a contrast-enhanced CT scan is useful to assess tumor extent and growth pattern in these rare variants. Among treatment alternatives, surgery sometimes complemented with radiotherapy is essential; constant follow-up is mandatory.

## Introduction

Adenoid cystic carcinoma (ACC) is the second most common malignant salivary gland tumor and accounts for about 10% of all salivary gland neoplasms and 30% of all minor salivary gland tumors [1]. Most malignant minor salivary gland tumors (MMSGT) arise in the oral cavity and oropharynx [2-3]. Out of 5,334 patients with MMSGT from the National Cancer Institute’s Surveillance, Epidemiology, and End Results (SEER) Program data, 229 (4.3%) ACCs were located in the larynx [3]. Incidence would be 0.005/100,000 individuals [4]. Eleven patients with ACC of the larynx and the trachea were previously reported over a 30-year period at a tertiary hospital of the University of California, Los Angeles (UCLA) [5]. Eleven tumors (2,4%) were found in the larynx/trachea among 450 patients with MMSGT reported from the Memorial Sloan Kettering Cancer Center [2]. We report an unusual case of an ACC which partially involved the cricoid cartilage and the adjacent superior aspect of the trachea.

## Case presentation

**Figure 1:**
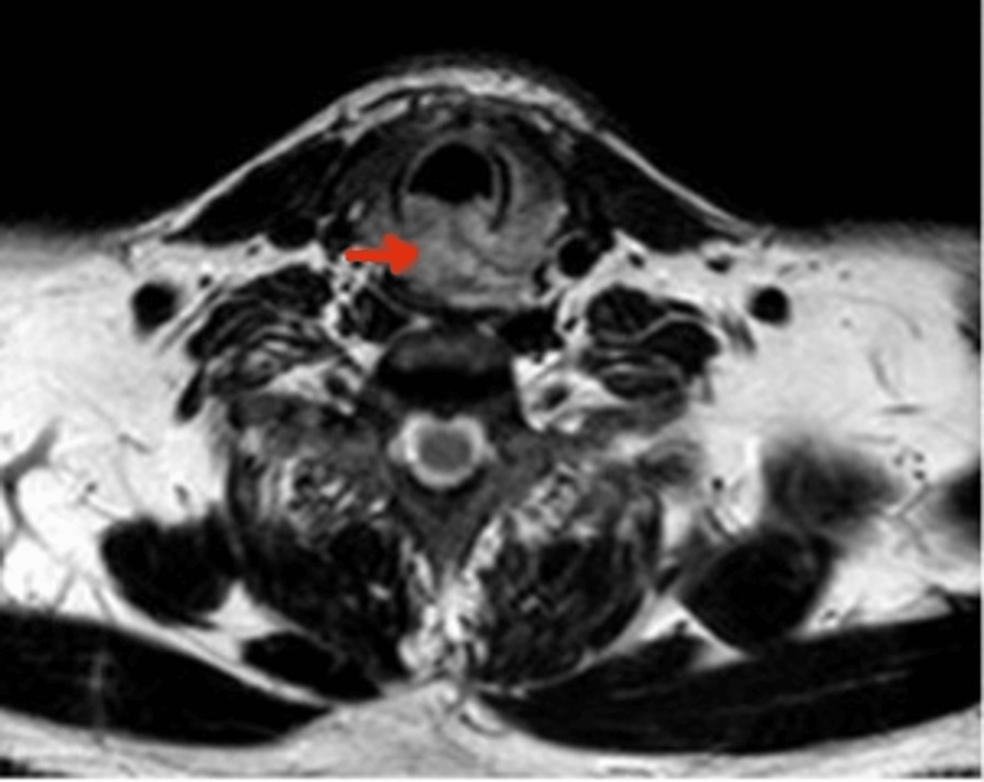
CT axial view showing a posterior endoluminal tumor that invades the posteroinferior perichondrium of the cricoid lamina

**Figure 2:**
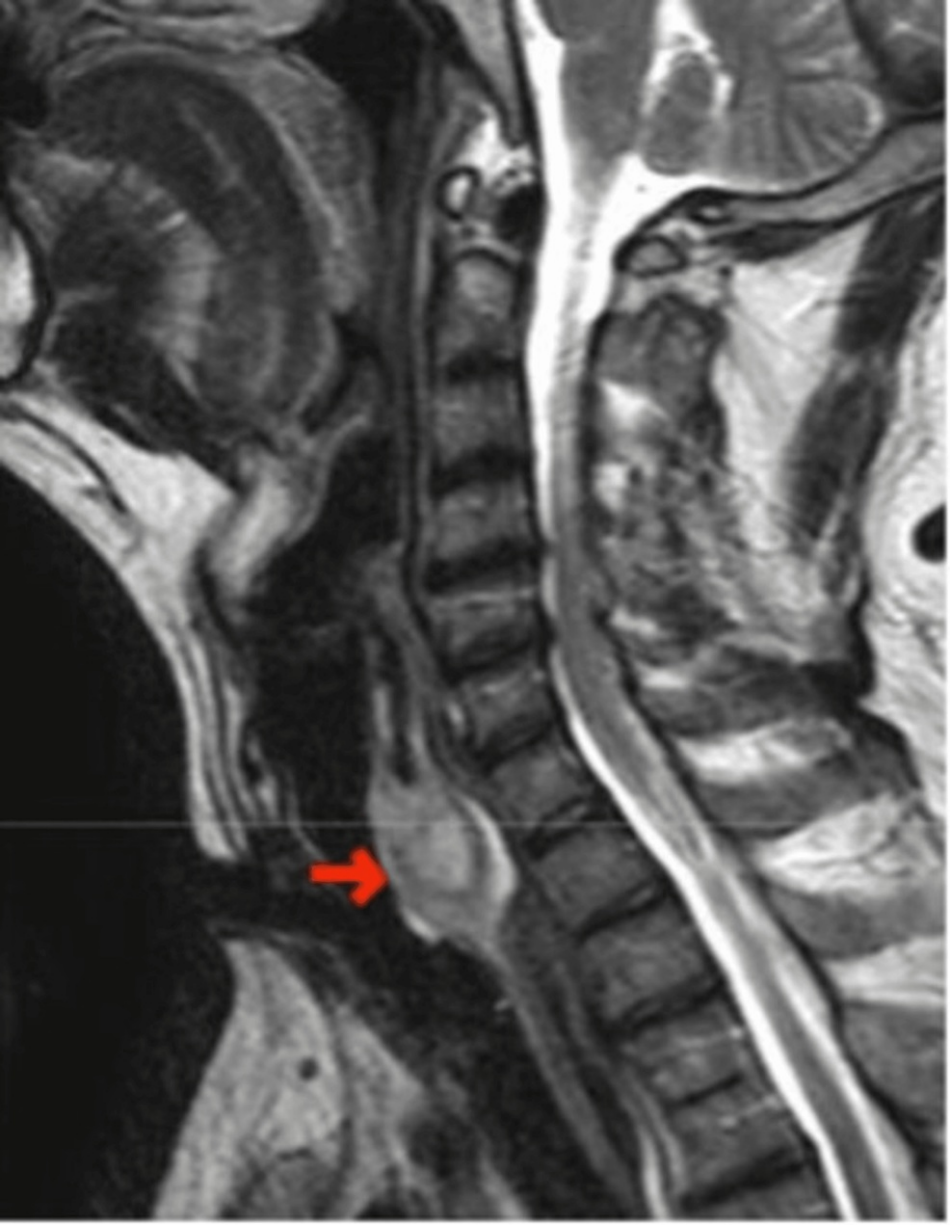
CT sagittal view of the posterior endoluminal tumor located at the upper part of the trachea that invades the posteroinferior perichondrium of the cricoid lamina and displaces the hypopharynx and esophagus

A 45-year-old otherwise healthy male was admitted to the emergency room because of two episodes of mild hemoptysis the same day. ENT and neck physical examinations were unremarkable. Upper gastrointestinal (GI) endoscopy was normal. A pharyngeal and laryngeal endoscopy showed normal endolarynx and congested proximal tracheal mucosa. A magnetic resonance imaging (MRI) revealed a posterior endoluminal tumor located at the upper part of the trachea that invaded the posteroinferior perichondrium of the cricoid lamina and displaced the hypopharynx and esophagus (Figures 1-2). A biopsy through a bronchoscopy procedure was not considered due to the possible risk of bleeding.

On the following day, he underwent a left-limited cervical surgical exploration and an intraluminal incisional biopsy through the tracheostomy space, performed by another surgical team. The pathological study reported an ACC. 

The lesion was classified as laryngeal T4aN0M0, stage IVA tumor, because it invaded the cricoid cartilage and extended to the trachea. A TNM classification has not been defined for the trachea in the latest edition of the American Joint Committee on Cancer (AJCC) Cancer Staging Manual [6]. But according to the TNM staging provided by Bhattacharyya, this lesion was also classified as a T4N0M0 tumor [7]. 

**Figure 3:**
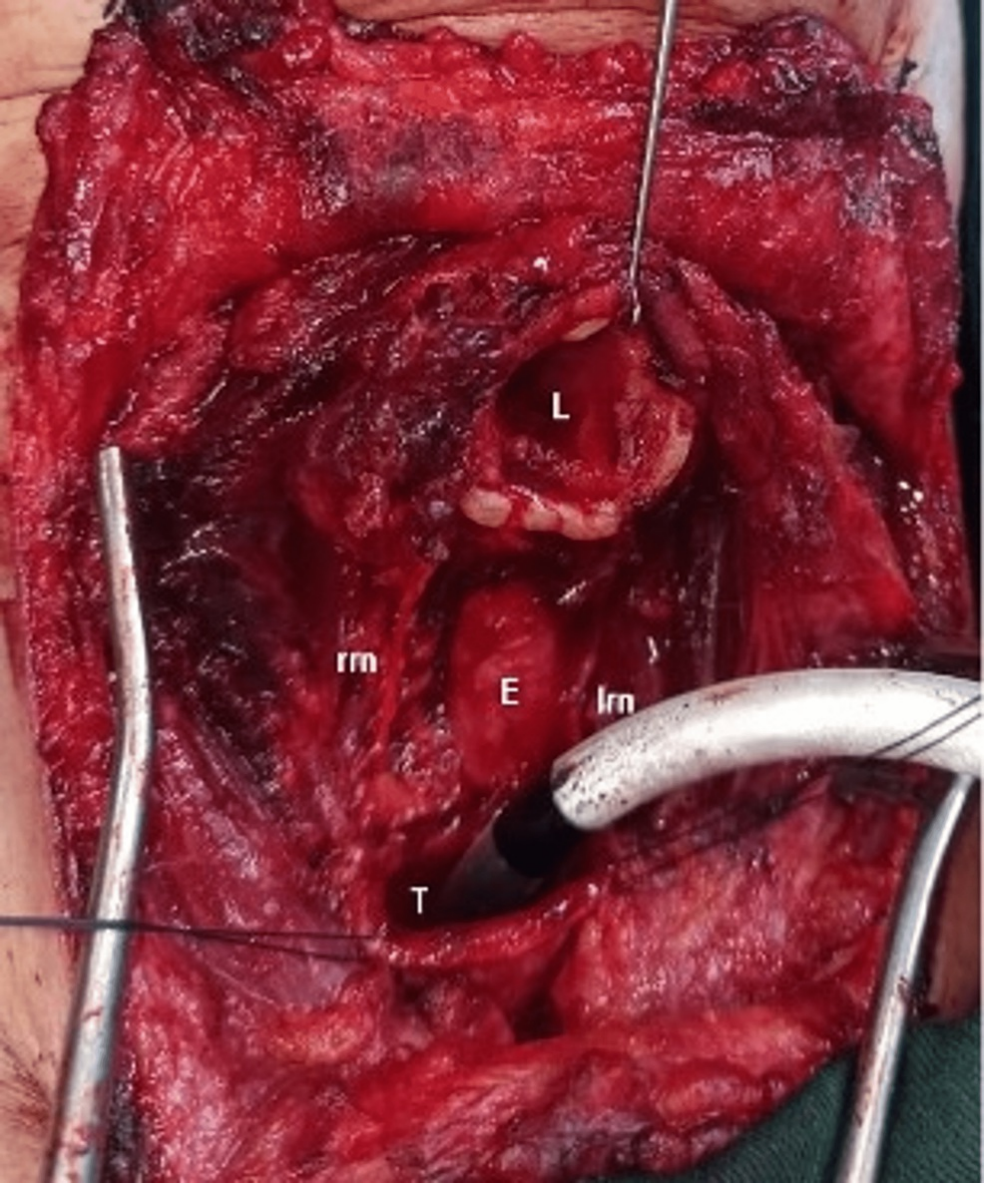
Surgical bed after cricotracheal resection L: larynx, E: esophagus, T: trachea, RRN: right recurrent nerve, LRN: left recurrent nerve.

The patient signed an informed consent before surgery. Total laryngectomy was an option, but invasion was very limited to the inferior border of the posterior cricoid lamina. Additionally, our young patient was reluctant to undergo this procedure and therefore, it was considered as the last option. A larger surgical exploration was performed, several days later, through a Kocher incision. A circular tracheal resection including the first four cartilaginous rings was carried out. This resection included a 2cm inferior part of the posterior cricoid lamina, the left thyroid lobe that was closely attached to the tumor, and the lymph nodes located under the thyroid isthmus. Both recurrent laryngeal nerves were dissected free but the left nerve was surrounded by the tumor (Figure 3).

**Figure 4:**
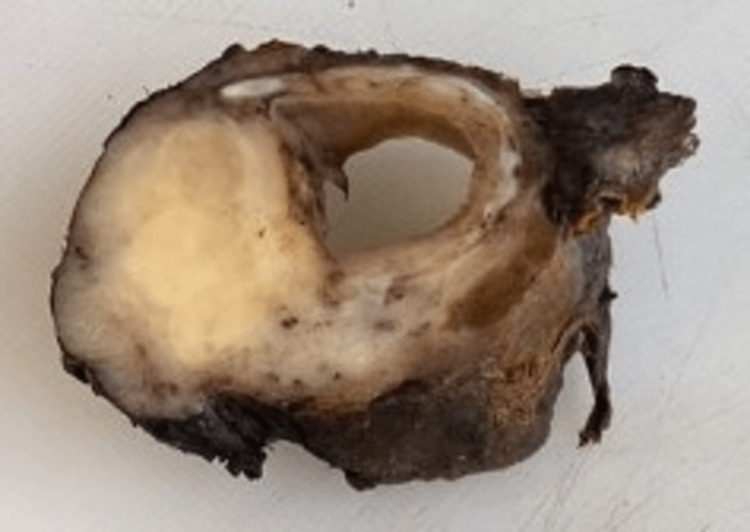
Circular cricotracheal resection specimen showing infiltrative tumor located in the posterior wall

Pathological macroscopic study of the specimen showed a lesion, 3cm in the cranio-caudal axis, 2.2cm in width and 1cm thick, located at the posterior wall of the cricoid cartilage and proximal trachea (Figure 4).

**Figure 5:**
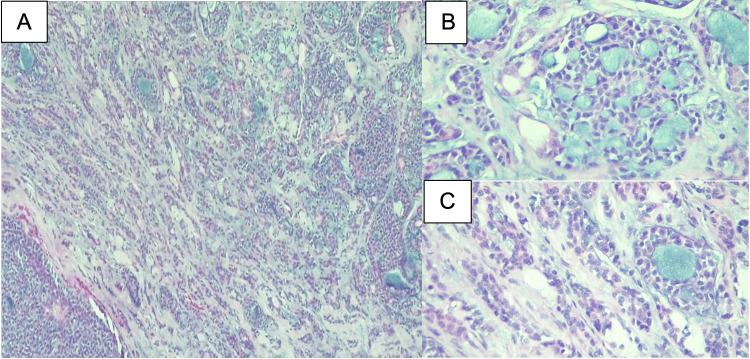
A: Low-power micrograph (hematoxylin and eosin (H&E) 10x). B and C: Low-power micrograph (H&E 40x): Pseudo glandular spaces filled with mucin and hyalinized surrounded by myoepithelial bland cells

Only the upper margin was compromised, but additional cricoid margin and the lower margin were free of tumor. Microscopically, the tumor showed the following patterns, in descending order: tubular, solid, cribriform, and trabecular. The tubular areas showed myoepithelial and ductal cells. The solid areas showed polyhedric cells, with clear cytoplasm and large and regular nuclei. The cribriform areas showed cystic spaces, with mucoid and hyaline content, surrounded by small, uniform, cuboid cells. Mitotic figures were scanty in all the patterns. Extensive perineural invasion was identified but there was no lymphovascular invasion (Figure 5). 

Postoperative evolution was uneventful except for a mild dysphonia due to left vocal cord paralysis. Postoperative 6600 cGy radiation therapy was administered. One and a half year after surgery, the patient remains with bilateral vocal cord mobility, normal speech and no evidence of tumor at a control neck MRI.

## Discussion

ACC is rarely located in the larynx due to the presence of a few accessory salivary glands inside its mucosa [8]. It represents <1% of all laryngeal tumors [8]. A systematic review by Marchiano et al. [9] in 2016 identified 120 cases of laryngeal ACC; most of them were located in the subglottic region, as in our case, [4]. On the other hand, 15% of tracheal malignancies are ACC [10]. In a study performed at the National Cancer Center of China, during a 50-year period, 191 cases of tracheobronchial ACC were reported [11]. In a recent systematic review of the literature [12], a total of 1,252 cases of tracheal ACC were identified.

It is important to perform a histopathological analysis of the sample before surgery. This would help identify low prognosis neoplasms such as basaloid SCC which would modify the whole management. ACC histologic patterns are cribriform (46,3%), solid (38,3%), and tubular (14,9%) [13]. According to its position inside the larynx, ACC may present with either dysphagia and hoarseness for supraglottic neoplasms, or dyspnea and stridor for subglottic neoplasms. Patients with tracheal tumors have cough with expectoration of scanty sputum and exertional dyspnea [12]. Hemoptysis without dyspnea as a first symptom, as in our patient, is unusual.

Most ACC are asymptomatic and present as non-ulcerated masses. Consequently, prompt identification of ACC is rare and maybe even attributed to asthma, amyloidosis, or other tracheobronchial diseases [14]. A biopsy for diagnosis can usually be performed by laryngoscopy or bronchoscopy but was omitted in our patient due to high bleeding risk. An open biopsy through a tracheotomy was preferred instead. Computed tomography (CT) is very useful to assess the primary tumor location, extra-luminal extension, and regional disease. Most patients with ACC,, as in our case, harbor T4 lesions at initial diagnosis, and 87.9% have no disease [4]. PET-CT could be performed to rule out distant metastases. However, there could be interpretation pitfalls in this imaging study [15]. Despite identifying metastasis, laryngectomy is still an appropriate alternative because ACC grows slowly, and quality of life must be optimized [16]. 

In this context, surgery is the pillar of treatment. Total laryngectomy might be appropriate for most subjects whereas partial laryngectomy should be reserved for selected patients with small and well-defined tumors [17]. In the review by Coca-Pelaz et al., [18] occult neck metastases in ACC patients compromising the larynx were low (15%) and no survival advantage was found in patients who underwent elective neck dissection. Chang et al. noticed a worse prognosis in lymph node-positive metastases; they suggested resection for stages T3 and T4 [13]. In this case report, no clinical lymph nodes were found.

For tracheal ACC, surgical resection followed by postoperative radiotherapy is the recommended treatment [19] and the long-term prognosis is favorable. In cases of incomplete resection, good overall survival can still be achieved with adjuvant radiotherapy. As per the study by Ran et al., [12], the most frequent treatments were surgery alone (40.9%), surgery with postoperative radiotherapy (36.4%), and radiotherapy alone (19.2%). Irradiation with carbon ions (C12) has shown promising results [10]. ACC might be sensitive to radiotherapy, but patients are not cured. Under these circumstances, radiotherapy with concurrent chemotherapy is a therapeutic option for tumors that cannot be resected [20]. Adjuvant radiotherapy improved prognosis in patients with positive lymph nodes, high grade, compromised surgical margins, and advanced stage [13-21]. Given the limited efficacy of chemotherapy for advanced tumors with distant metastases [22], a more personalized approach is of utmost importance. So significant efforts are being undertaken to improve outcomes with biomarker-driven research and subtype-specific targeted therapy [23]. 

After an average follow-up time of 54.0 months, 55.3% of laryngeal ACC patients, treated with surgery and radiotherapy, were alive with no evidence of disease, according to Marchiano’s systematic review [9]. Five-year disease-free survival was 73.7% for patients treated with surgery in Dubal’s review [4]. According to the review by Ran et al. [12] of tracheal ACC, the five- and ten-years survival rates of patients treated with surgery alone and surgery with postoperative radiotherapy were 86.4%, 55.6% and 97.3%, 44.4%, respectively.

## Conclusions

ACC is a rare malignancy of the larynx and trachea. A contrast-enhanced CT scan allows the assessment of the development and involved anatomical regions of the neoplasm. Evaluation by histopathology is important to correctly characterize the sample. Among treatment alternatives, surgical resection with wide safe margins is the optimal therapeutic approach, eventually complemented with radiotherapy in case of positive margins. Finally, even if we are certain that a prolonged follow-up is crucial to prevent recurrence, our patient remains in good clinical condition one-and-a-half years after initial treatment. 
